# The Unique Dorsal Brood Pouch of Thermosbaenacea (Crustacea, Malacostraca) and Description of an Advanced Developmental Stage of *Tulumella unidens* from the Yucatan Peninsula (Mexico), with a Discussion of Mouth Part Homologies to Other Malacostraca

**DOI:** 10.1371/journal.pone.0122463

**Published:** 2015-04-22

**Authors:** Jørgen Olesen, Tom Boesgaard, Thomas M. Iliffe

**Affiliations:** 1 Natural History Museum, University of Copenhagen, Universitetsparken 15, 2100 Copenhagen Ø, Denmark; 2 Department of Marine Biology, Texas A & M University at Galveston, Galveston, Texas, 77553–1675, United States of America; Laboratoire de Biologie du Développement de Villefranche-sur-Mer, FRANCE

## Abstract

The Thermosbaenacea, a small taxon of crustaceans inhabiting subterranean waters, are unique among malacostracans as they brood their offspring dorsally under the carapace. This habit is of evolutionary interest but the last detailed report on thermosbaenacean development is more than 40 years old. Here we provide new observations on an ovigerous female of *Tulumella unidens* with advanced developmental stages in its brood chamber collected from an anchialine cave at the Yucatan Peninsula, which is only the third report on developmental stages of Thermosbaenacea and the first for the genus *Tulumella*. Significant in a wider crustacean context, we report and discuss hitherto unexplored lobate structures inside the brood chamber of the female originating at the first (maxilliped) and second thoracic segments, which are most likely modified epipods, perhaps serving as gills. At the posterior margin of carapace of the female are rows of large spines preventing the developing stages from falling out. The external morphology of the advanced developmental stages is described in much detail, providing information on e.g., carapace formation and early limb morphology. Among the hitherto unknown structures in the advanced developmental stages provided by this study are the presence of an embryonic dorsal organ and rudimentary ‘naupliar processes’ of the second antennae. Since most hypotheses on crustacean (and malacostracan and peracaridan) relationship rest on external limb morphology, we use early limb bud morphology of *Tulumella* to better establish thermosbaenacean limb homologies to those of other crustaceans, which is a necessary basis for future morphology based phylogenetic considerations.

## Introduction

Thermosbaenacea is a small taxon of malacostracan crustaceans (about 34 species) that inhabit subterranean mostly marine waters [1]. Among malacostracan crustaceans only Thermosbaenacea brood their offspring dorsally under an enlarged carapace, superficially similar to what is seen in certain branchiopod crustaceans (water fleas), ostracods, and ascothoracid thecostracans (e.g., [2]). This, among malacostracans, unusual habit is of evolutionary interest since all presumed close relatives to the Thermosbaenacea brood their offspring in a ventral marsupium (other peracarids), between the thoracopods (leptostracans), attached to pleopods (Pleocyemata), or they have free larvae only (e.g., krill and dendrobranchiate shrimps) (see reviews in [3]). The dorsal brood pouch was one of the reasons for treating the Thermosbaenacea as ‘Pancarida’, a taxon distinct from the Peracarida but closely related to it [4]. Despite the evolutionary interest of brooding and development in Thermosbaenacea, most information is based on two species only, and significant primary data have not appeared in more than 40 years. Dorsal type of brooding and the data on developmental stages was first reported for *Tethysbaena argentari* from an Italian cave system (at Monte Argentario) [5–7]. Shortly after dorsal brooding was found also in *Thermosbaena mirabilis* (inhabiting hot springs in Tunesia) [8], confirming that this developmental habit is indeed general for Thermosbaenacea, and much information on the same species has later appeared including that brood-carrying females swim on their backs and that eggs are transferred to the dorsal brood pouch in thin-walled sacs secreted from the vaginal papillae of 6^th^ thoracopods [9–11]. Ovigerous females with enlarged carapaces have been reported for many other species [12, 13], but the development has not been studied in further detail.

Here we provide new observations on an ovigerous female and on an advanced developmental stage of *Tulumella unidens* collected from an anchialine cave at the Yucatan Peninsula, which is only the third report on the ontogeny of Thermosbaenacea and the first for the genus *Tulumella*. We describe the external morphology of an advanced developmental stage and report hitherto unexplored structures inside the brood chamber (large lobes) and on the posterior margin of the carapace (large spines) of the female. Probably due to a specialised feeding mode (scraping, brushing and pushing, but see [14]) in Thermosbaenacea involving only the cephalic appendages [15], some uncertainty exist concerning the precise homologies to the mouth parts of other crustaceans (see [4, 12, 15]. Mouth part homologies are important when considering phylogeny and their study has a long tradition (e.g., [16–18]). Since the precise phylogenetic position of the Thermosbaenacea within the Malacostraca is still debated [19–22], we attempt to use the new developmental data to provide a hypothesis for mouth part homologies of the Thermosbaenanacea with other crustaceans, which is a necessary basis for future morphology based phylogenetic considerations.

## Material and Methods

**Fig 1:**
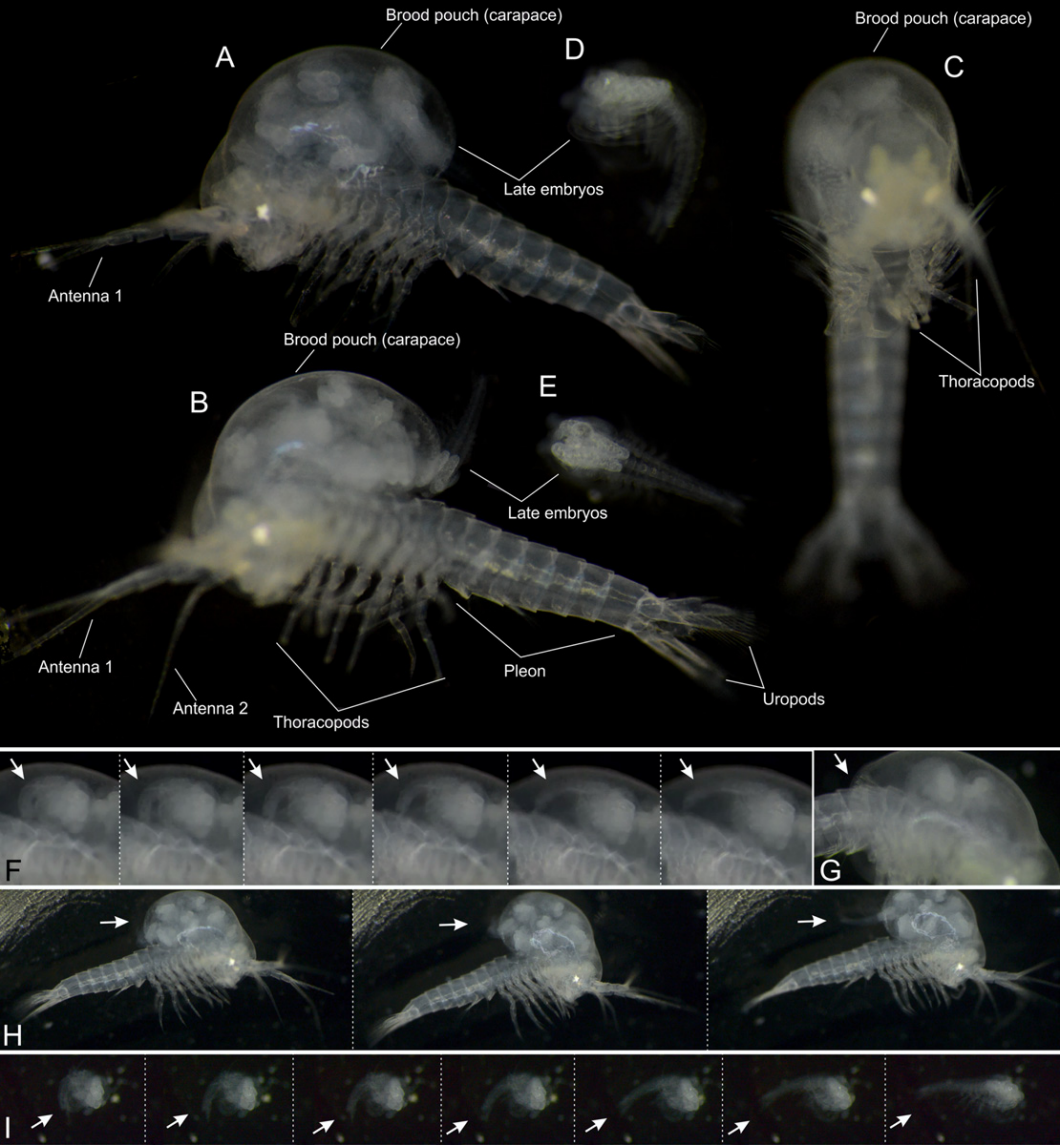
*Tulumella unidens* (Thermosbaenacea), photographs and video-sequences of live ovigerous female and advanced developmental stages. A, lateral view. B, lateral view. C, frontal view. D, lateral view of newly released specimen. E, dorsal view of advanced stage. F, close-up of advanced stage in brood pouch with ventrally flexing body (arrows). G, close-up of ovigerous female showing posterior opening of brood pouch (arrow). H, ovigerous female in the process of releasing an advanced developmental specimen (arrow). I, newly released specimen with ventrally flexing body (arrows).

**Fig 2:**
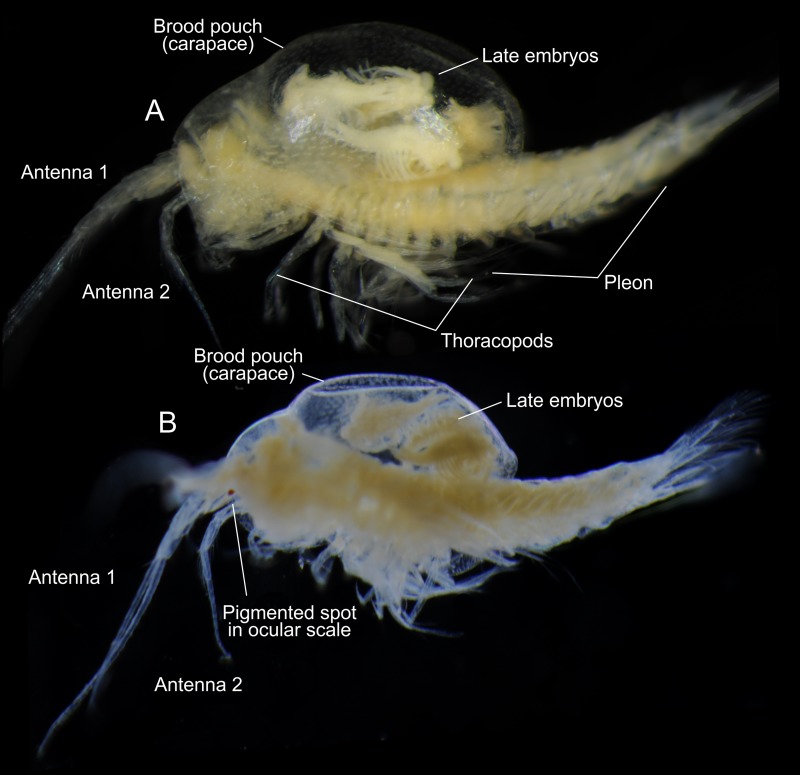
*Tulumella unidens* (Thermosbaenacea), photographs of ovigerous female (fixed specimen). A, with reflected light. B, with transmitted light.

**Fig 3:**
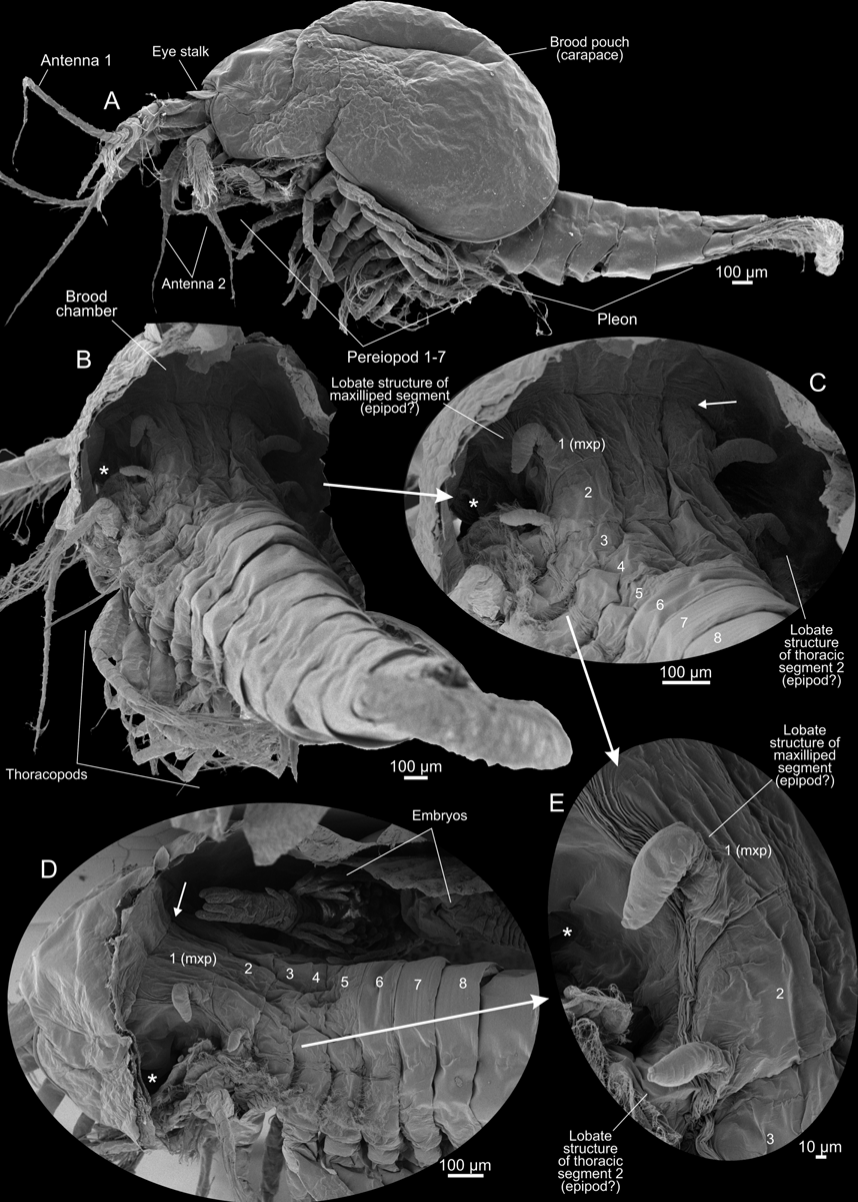
*Tulumella unidens* (Thermosbaenacea), scanning electron micrographs of ovigerous female. A, lateral view. B, posterior view with part of brood pouch/carapace removed. C, close-up of dorsal part of thorax after removal of brood pouch. D, close-up of dorsal part of thorax after removal of brood pouch. E, lobate structures (epipods?) of thoracic segments 1 (maxillipedal) and 2.

**Fig 4:**
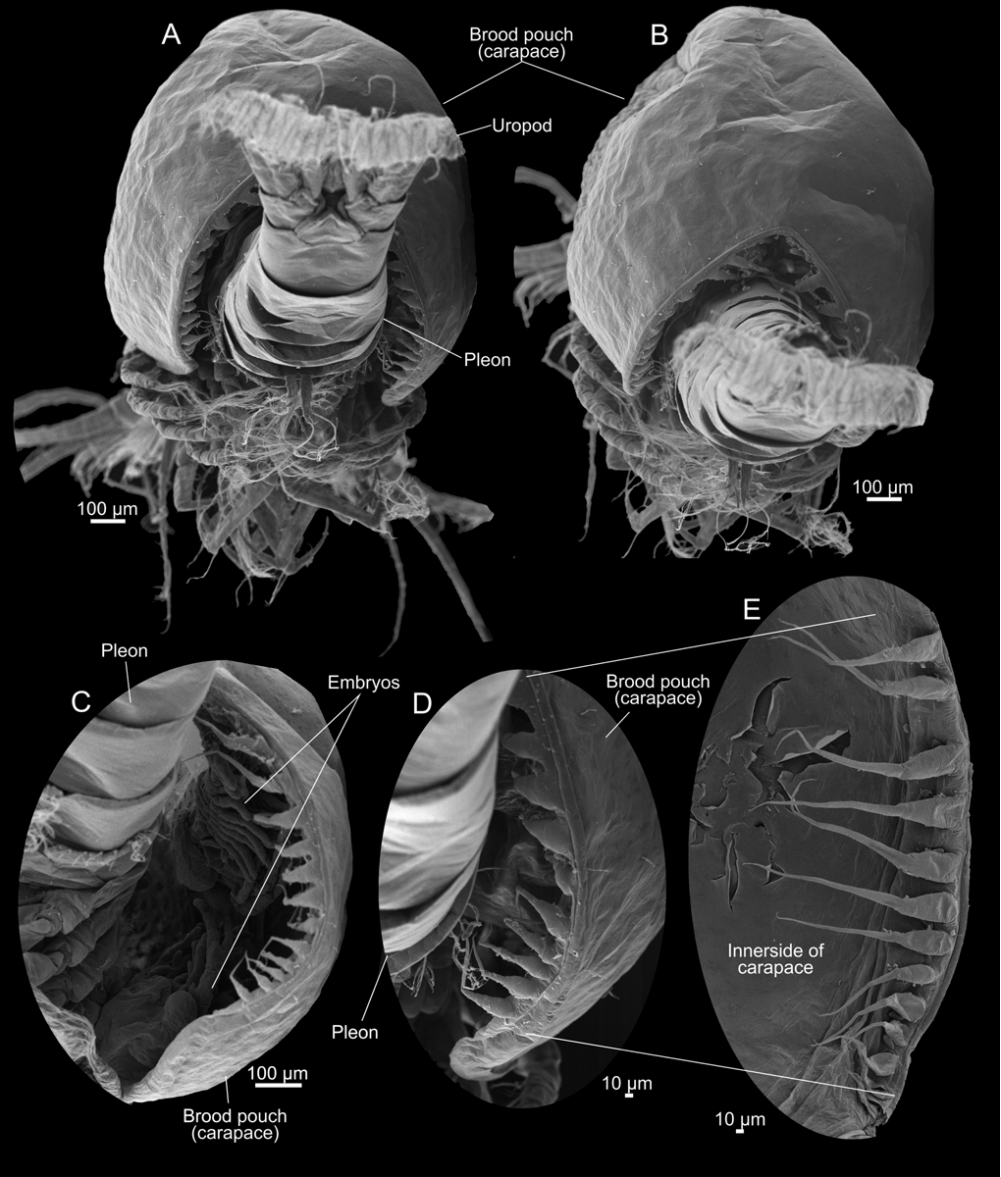
*Tulumella unidens* (Thermosbaenacea), scanning electron micrographs of ovigerous female. A, B, posterior view showing uropods, telson, pleon and posterior margin of brood pouch (carapace). C, D, right side posterior margin of brood pouch (carapace) showing large marginal spines and developmental stages inside. E, close-up of marginal spines.

**Fig 5:**
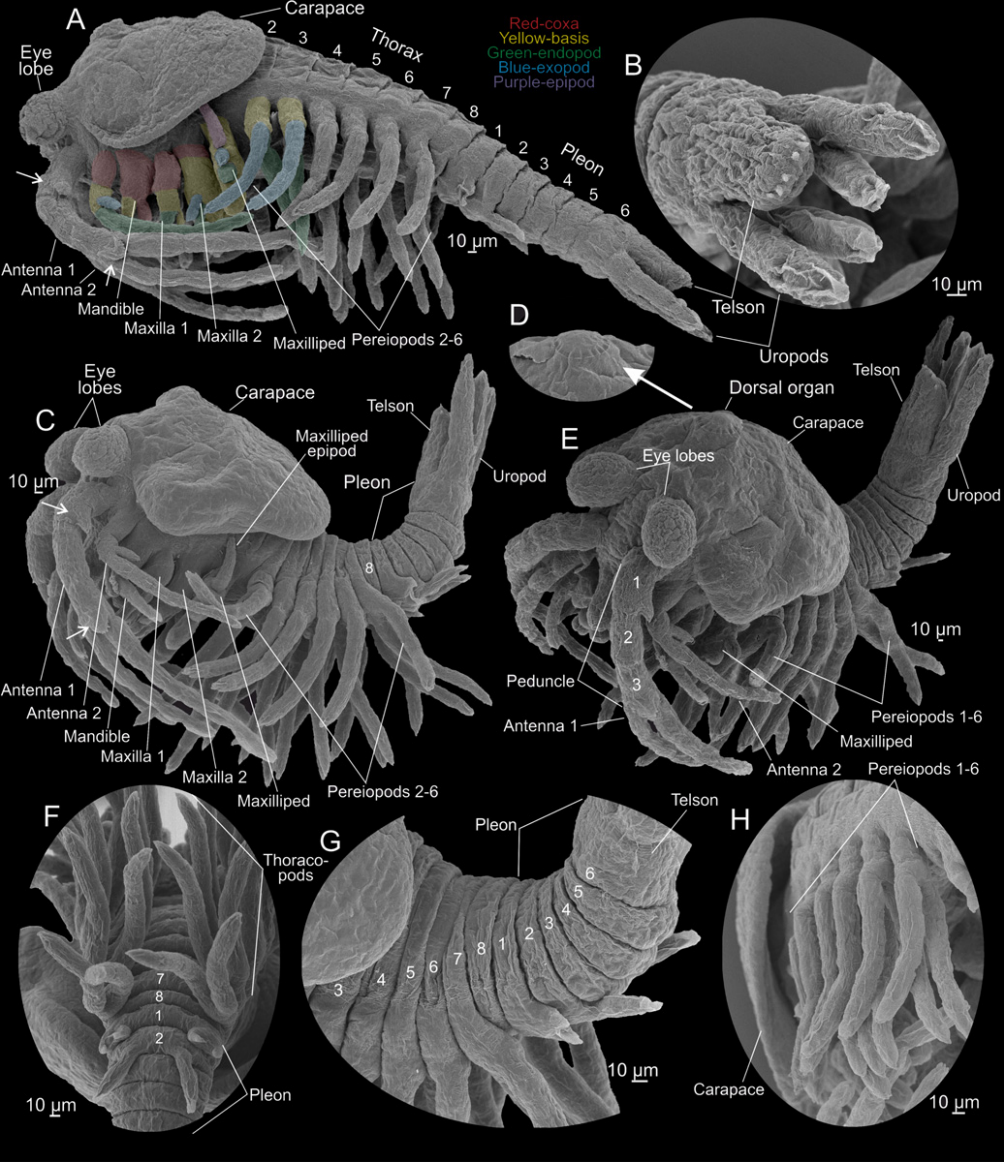
*Tulumella unidens* (Thermosbaenacea), scanning electron micrographs of late developmental stages removed from brood pouch of ovigerous female. A, whole view from lateral. B, telson and uropods. C, whole view from lateral. D, embryonic dorsal organ. E, whole view from antero-lateral. F, thoracopods and pleopods from ventral. G,pleon and part of thorax seen from lateral. H, postero-lateral view of thoracopods (pereiopods) of left side. Colour code: Red-coxa, yellow-basis, green-endopod, blue-exopod, purple-epipod.

**Fig 6:**
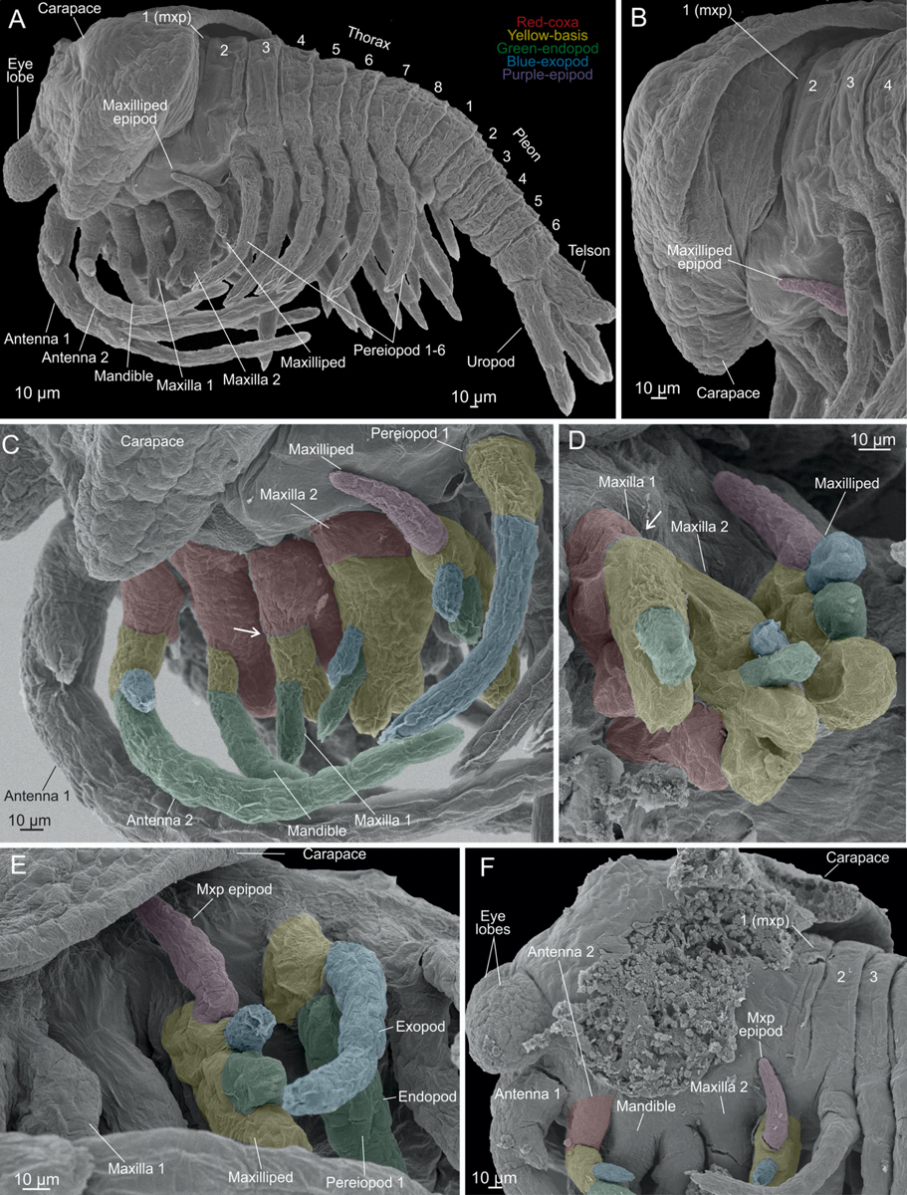
*Tulumella unidens* (Thermosbaenacea), scanning electron micrographs of late developmental stages removed from brood pouch of ovigerous female. A, whole view from lateral. B, posterior margin and attachment region of carapace seen from postero-lateral. C, lateral view of cephalic appendages (antenna 1 to maxilliped) and pereiopod 1 of left side. D, buds of maxilla 1, 2 and maxilliped seen from antero-lateral. E, maxilliped and pereiopod 1 seen from lateral. F, lateral side of cephalon with part of carapace removed which reveals its zone of attachment. Colour code: Red-coxa, yellow-basis, green-endopod, blue-exopod, purple-epipod.

**Fig 7:**
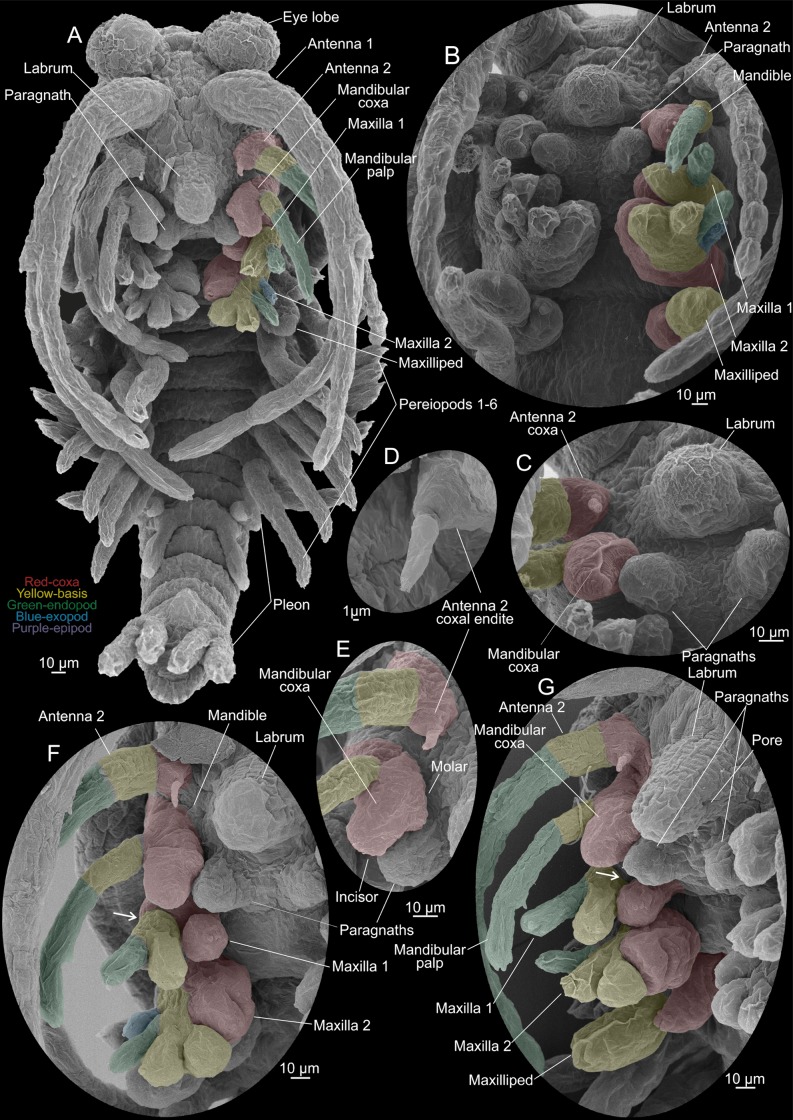
*Tulumella unidens* (Thermosbaenacea), scanning electron micrographs of late developmental stages removed from brood pouch of ovigerous female. A, whole view from ventral. B, close-up of cephalic appendages from ventral. C, labrum, paragnaths, and proximal parts of antenna 2 and mandible. D, coxal endite (= ‘naupliar process’) of antenna 2. E, proximal parts of antenna 2 and mandible. F, median view of cephalic appendages (antenna 2 to maxilla 2). G, median view of cephalic appendages (antenna 2 to maxilliped). Colour code: Red-coxa, yellow-basis, green-endopod, blue-exopod, purple-epipod.

During exploration of several anchialine cave systems at the Yucatan Peninsula, Mexico June 16–26, 2013, an ovigerous female was collected at the Cenote ‘Temple of Doom’ and brought alive to the lab in Tulum. The specimen was kept alive for several hours while it was photographed with a Nikon D800 fitted to an Olympus dissecting scope with an a ‘LM Scope’ adapter (Fig 1) using two wireless remote flashes. A number of HD quality movies were recorded with the same camera settings using 60 fps with a variety of LED light sources. After initial observation and documentation the specimen was fixed in 2% buffered glutaraldehyde, brought to Copenhagen, where further photos were taken using both reflected (Fig 2A) (setup as above) and transmitted light (Fig 2B). To enhance resolution of the image in Fig 2A several photos (about 5) were taken at different focus planes and combined in Zerene Stacker 1.04. After this the developing stages were removed from the brood pouch of the female and all material was dehydrated in a graded series of ethanol and acetone and critical point dried. The specimens were mounted for SEM and coated with a mixture of palladium and platinum and examined with a JEOL JSM-6335-F (FE) (Figs 3–7). After initial observation in SEM the ovigerous female went through a number of ‘post-dissections’ with subsequent re-sputtering to reveal structures inside the brood chamber (Fig 3B–3E), and also the developing specimens in some cases underwent additional dissection after having been critical point dried and mounted on a stub (Fig 6F). The photos were handled in standard graphics programs such as Adobe Photoshop and the CorelDraw package, and a few sequences of videos were grabbed in GOM Player 2.2 (freeware) and shown here in Fig 1F–1I. A number of video sequences of live specimens are shown as additional material (S1–S2 Videos). Collecting permit was issued by SEMARNAT (Secretaría de Medio Ambiente y Recursos Naturales), Mexico City to Thomas M. Iliffe via a permit to Dr. Fernando Álvarez Noguera (Universidad Nacional Autónoma de México) (FAUT-0104). The landowner gave permission to conduct the study on the site. The field studies did not involve protected species. Texas A&M University is an AAALAC, International accredited facility which conducts all vertebrate animal activities involving research, teaching, and testing according to the regulations set forth by the Animal Welfare Act, the Guide for the Care and Use of Laboratory Animals, and the Public Health Service Policy. The work conducted by Dr. Iliffe and colleagues involved the use of invertebrates which are not specifically covered by the regulations previously listed but are also afforded the highest standard of care when used in research, teaching, or testing. No work on living specimens was conducted in Europe. All procedures in this investigation complied with international and institutional guidelines, including the guidelines for animal welfare.

## Results

### Ovigerous female, info based on videos of live specimen (Fig 1)

The ovigerous female has a large globular carapace which is modified to a brood pouch holding 12 advanced developmental stages (Figs 1–3). The ovigerous female was observed alive, photographed, and filmed in a Petri dish in the lab for a couple of hours before fixation (Fig 1). These observations reveal a clear tendency for the ovigerous female to lie on its side or its back while the thoracopodal exopods are beating (S1 Video), probably because of top-heaviness due to the very large dorsal brood pouch. Also when the female occasionally swam in the Petri dish, the brood pouch was often directed downwards. The developing specimens in the brood pouch performed feeble movements in particular by flipping slowly the hind body up and down (Fig 1F and S2 Video). After having been kept for a couple of hours in the lab, the ovigerous female started slowly to flip its body up and down a number of times causing the posterior opening of the brood pouch to become larger resulting in the release of a couple of developmental stages (Fig 1H and S3 Video). A row of long setae/spines (described further below) along the posterior margin of the brood pouch normally would serve to keep the developmental stages inside the brood pouch (Figs 1G and 4, S4 Video), but when the posterior opening of the brood pouch becomes large enough, they are unable to do this. Outside the brood pouch the advanced developmental stages continued flipping the hind body slowly up and down and also performed weak limb movements, but they were incapable of moving around (Fig 1I, S5 Video). Probably the release of these specimens was caused by non-natural conditions in the lab such as higher temperatures, light, and handling by the observer (forceps, pipettes, etc.).

### Ovigerous female, description of morphological aspects related to brooding (Figs 3 and 4)

The posterior margin of the brood pouch (carapace) in the ovigerous female reaches slightly further posteriorly than the border between the thorax and the pleon (Figs 2 and 3A). Removal of a part of the carapace revealed that it dorsally is an extension of the region anterior to the maxilliped (thoracic 1) segment (Fig 3B–3D), and it is likely that the transverse suture (arrows in Fig 3C and 3D) where the carapace meets the dorsal side of the animal body represents the posterior margin of the cephalon, i.e, the rear of the maxilla 2 segment. But it may also be that the rather wide area marked ‘mxp’ on Fig 3C and 3D represents more than the maxilliped segment, in which case the carapace does not extend exactly from the rear of the cephalon.

The posterior margin of the brood pouch (carapace) forms a V-shaped opening with each side of the ‘V’ fringed with long setae-like projections serving to keep the developmental stages inside the brood pouch (Figs 1G, 1H, 4A and 4D, S4 Video). There are about 15 of these projections on each side getting gradually larger dorsally (4E).

Partly removal of the carapace revealed the presence of two pairs of short, lobate structures (probably epipods, but see Discussion) attached dorso-laterally at the body (Fig 3B–3E), one pair at the maxilliped segment, another pair at the second trunk segment; the pair at the maxilliped segment is clearly separate from the maxillipeds, the other pair is closer to the thoracopods The lobate structures of the maxilliped segment are the largest, about 120 μm long, about 4 times as long as wide, and bent approximately 90° with the tip pointing away from the mid-axis of the animal. The pair of lobes on the second thoracic segment has practically the same morphology but are slightly smaller and more straight of shape. All lobes are devoid of setation but have some weak furrows externally. A couple of non-ovigerous specimens were examined for the eventual presence of such lobes, but none were found.

### Advanced developmental stages (Figs 1D, 1E and 1I, 5–7)

The ovigerous female carried 12 advanced developmental stages. No membrane is covering the specimens. As mentioned above, when placed in the brood pouch, the live specimens move various limbs feebly and flex the pleon ventrally (Fig 1F, S2 Video), and the same behavior was seen in a few specimens that fell out during handling in the lab (see above) (Fig 1I, S5 Video). Preserved developmental stages never have the pleon flexed ventrally as in the live specimens; rather the body is stretched more or less out or even with the pleon flexed dorsally (Figs 5 and 6). Length is approximately 700–800 μm from eye lobe to tip of the uropods. All specimens were developed to approximately the same degree and the few minor differences may be artificial due to specimens reacting differently on fixation or critical point drying. The specimens correspond approximately to stage VIII or IX of *Thermosbaena mirabilis* (see [10, 23]).

The general shape and appearance, including number of body segments and limbs of the advanced developmental stages, is very much like that of adults, but many structures such as limb parts are less developed and setation has not yet appeared making limb segmentation etc. simpler to follow (Figs 5–7). The body is roughly divided in a cephalic, a thoracic, and a pleon portion (Figs 5A–5C, 5G and 6A). The thorax has eight free segments, but thorax segments 1 (mxp segment) and 2 are only separated from each other dorsally (Fig 6A, 6B and 6F); the pleon has six free segments; all body segments (thorax and pleon) are approximately equally narrow, with the exception of thorax segment 2 and pleon segment 6, which are wider. The cephalic portion carries eye lobes, an early carapace, antennae 1 and 2, mandibles, maxillae 1 and 2, maxillipeds, labrum, and paragnaths. The maxilliped segment seems to be fused with the remaining cephalic portion at least laterally since no segmental furrow separates the ‘maxilla 2 area’ from the ‘maxilliped area’ here. The situation dorsally is more uncertain since the carapace seem to extend exactly where such a furrow would have been (carapace described further below) (Fig 6F). Also the thorax segment 1 appears fused laterally (but not dorsally) with the cephalic portion. Thorax segments 2–7 each carry a pair of pereiopods (6 pairs in total); thorax segment 8 is devoid of limbs (Figs 5A and 6A). Pleon segments 1 and 2 each carries a pair of small appendages; pleon segments 3–5 lack limbs; pleon segment 6 carries a pair of uropods; posteriorly is the telson (Figs 5 and 6A).

The eye lobes are a pair of large globular, slightly elongate structures placed anteriorly at the cephalon above the first antennae and directed slightly downwards distally (Figs 5A–5C, 5E, 6A, 6F and 7A). A free carapace lobe is extending both laterally and at the rear of the cephalic portion overhanging the basal parts of the head appendages and dorsally extending to about thorax segments 2–4 (Figs 5A, 5C, 6A and 6B). In a specimen where the carapace has been partly removed, and its zone of attachment to the cephalon has been revealed, it is seen that dorsally only the maxilliped (thorax 1) segment is free, while a larger portion of the cephalon is free laterally, possibly also a part of the maxilla 2 segment (Fig 6F). Dorsally, approximately in the position of the second antennae and the mandibles, is a circular dorsal organ (Fig 5D and 5E). The first antennae, which insert antero-ventrally at the cephalic region are very long extending to the pleon region (Figs 5A, 5C, 6A and 7A). Each first antenna consists of a large peduncle which extends into two branches of approximately similar length as the peduncle. The peduncle is subdivided into three portions of which the proximal is the widest (Fig 5E, marked 1–3). The proximal portion extends into a short, spine-bearing bud laterally. The distal portion of the peduncle also carries a short spine-bearing bud. The anterior branch of the first antenna, which is the shortest, is sub-divided in about six weakly indicated tubular segments (Fig 5A and 5C). The longer posterior branch is subdivided into about 7–8 weak segments. Each second antenna is composed of a short broad portion interpreted as a coxa carrying a spine medially, which is the rudiment of a ‘naupliar process’, followed by a tubular limb portion which is interpreted as a basis (Figs 6C and 6F, 7). The basis continues medially into a long, slender endopod which is subdivided into 7–8 primordial segments, and a short, unsegmented exopod laterally (Fig 6C). The mandible is composed of broad, swollen coxa, a slender basis, and a two-segmented endopod (Figs 6C, 7A, 7C, 7E and 7G). The coxa consists of a small lateral part and a larger, elongate median part, which will become the gnathal edge in the adult (Fig 7E and 7F). The distal part of the gnathal edge will develop into a long incisor (see [12, 24, 25]). In the ventral midline of the specimen, in the centre between the second antennae and the mandibles, a conical and slightly posteriorly pointed labrum is present (Fig 7); on each lateral face of the labrum is a small pore (Fig 7G). A pair of widely separate paragnath humps is present posteriorly to the labrum (Fig 7B, 7C, 7F and 7G). The first maxillae limb buds are composed of a short coxa basally carrying a distinct globular lobe medially which will become the coxal endite in the adult, a short basis medially extending into an endite, and a slender limb portion distally interpreted as an endopod (Figs 6C, 6D, 7F and 7G). The second maxillae limb buds are flattened, rounded multilobate structures (Figs 6C, 6D, 7A, 7B,7F and 7G). Proximo-medially is a large lobe, directed somewhat anteriorly, which in some specimens is divided in a large basal part and a smaller distal part (Fig 7F and 7G). Based on serial homology to the first maxilla and the mandible, and on the fact that the basal and distal parts of this large lobe is only weakly subdivided from each other, the combined structure is interpreted as the median part of the coxa (see Discussion). The following two endites are placed medio-distally at the limb bud and are interpreted as two basipodal endites (Figs 6D,7B, 7F and 7G). The endopod is represented by a tubular lobe attached latero-distally at the limb bud (Figs 6C, 6D, 7B, 7F and 7G). The exopod is a slightly smaller tubular lobe attached anteriorly at the limb bud (Fig 6C and 6D). The maxilliped limb bud is medially divided into a proximal enditic lobe being a coxal endite and a large medio-distal lobe being a basipodal endite (Fig 7B and 7G). Lateral to the endite is a small, distal lobe representing the endopod, and lateral to this a slightly larger lobe representing the exopod (Fig 6C–6E). Laterally at the limb bud is a long, dorsally directed lobe which represents the epipod (Fig 6). Pereiopods 2–6 are practically identical to each other consisting of a short stem weakly subdivided into coxal and basipodal portions of approximately similar sizes (Fig 5A, 5C,5F and 5H). Laterally the basipodal portion of the thoracopods carries a laterally bend exopod, about 4 times the length of the limb stem, and divided into one rather long and distinct proximal portion and some smaller and more unclearly defined portions distally. The endopod is about 5 times the length of the stem and weakly subdivided into a number of portions most pronounced distally, where the limbs are also bent slightly medially foreshadowing the condition in the adult. The last thorax (no 8) segment bears no limbs (Fig 5A and 5G). Pleopods 1 are small, broad limb buds each terminating in a pair of lobes representing the endopod and the exopod (Fig 5F). Pleopods 2 are small, slender uniramous buds consisting of an endopod and a small basipod fused to each other (Fig 5F). Pleon segments 3–5 are devoid of limbs (Fig 5A and 5G). Pleon segment 6 carries a pair of long posteriorly directed uropods (Fig 5A–5C, 5E and 5G). The length of the uropods are approximately 2/3 the length of the pleon. Each uropod consists of a short stem carrying an endopod and exopod twice the length of the stem. The exopod, which is the outer ramus, is slightly longer than the endopod and is divided in a basal portion and a slightly shorter distal portion. Dorsal to the uropods is an elongate, fleshy telson being rounded terminally and carrying six incipient setae arranged in a row along the margin (Fig 5B).

## Discussion

### Dorsal brooding of and ventrally flexed embryos in Thermosbaenacea

We report the finding of a female of *Tulumella unidens* (Thermosbaenacea) with a carapace modified as a dorsal brood pouch holding 12 advanced developmental stages (Figs 1 and 2). Among malacostracan crustaceans this unique type of brooding has earlier only been described in some detail for *Tethysbaena argentarii* [5–7] and later in more detail for *Thermosbaena mirabilis* [8–11]. As the last treatment was more than 40 years ago, the finding of yet another example of an ovigerous female, representing a third genus, *Tulumella*, which allowed for the study by SEM, was considered important. The 12 developmental stages differed from each other only in minor details so they are assumed to belong to approximately the same developmental stage. The development of *Thermosbaena mirabilis* has been reported to pass through 10 stages before release from the brood pouch, the six first of these being still within the egg membrane [10]. The stage examined by us of *Tulumella unidens* corresponds approximately to stage 8 or 9 of *Thermosbaena mirabilis*, which is the third stage after the egg membrane has been shed and the second last stage still in the brood pouch of the female. From the two other species where developmental information is available, it is known that the embryos become ventrally flexed when they elongate and the space within the egg membrane becomes limited. For *Thermosbaena mirabilis* it has been reported that the embryos become stretched out after the egg membrane has erupted [10]. The developing specimens of *Tulumella unidens* studied here was not enclosed in any egg membrane, and, while they were still alive, they were capable of performing movements both inside and outside the female’s brood pouch (some specimens fell out during handling), and especially the hind body was repeatedly flexed ventrally (Fig 1F and 1I, S5 Video). A ventrally flexed hind body, or growth zone, as that in thermosbaenaceans is seen in most malacostracan embryos and has been suggested to be plesiomorphic [20]. In some ingroup peracarids, e.g., Cumacea, Isopoda, Mictacea, Tanaidacea (see references [20]), and recently also found in Speleaogriphacea [23], the embryonic hind body is flexed dorsally, which is generally considered apomorphic. Thermosbaenacea are at times grouped with Mictacea and Spelaeogriphacea (e.g., [19]), but the difference in the orientation of the hind body within the egg membrane (ventrally *versus* dorsally flexed) is a significant character separating the Thermosbaenacea from the two. Another character setting the Thermosbaenacea aside is the presence of dorsal brooding (see Figs 1 and 2), which is significantly different from the ventral brooding in a marsupium seen in all other peracarids. The presence of embryos/developmental stages brooded dorsally under the carapace of the female is remarkable in a malacostracan context, and the evolutionary pathway leading to this unusual type of brooding remains to be clarified. The following observations may provide a hint. For another thermosbaenacean, *Thermosbaena mirabilis*, it has previously been noted that prior to the transfer of the embryos to the dorsal brood chamber these are carried in a pair of dorsolateral egg pouches, which are attached to the basal parts of thoracopods 6 in a position where the eggs/embryos emerge (see drawings in [9, 23]). Later these pouches degenerate and the embryos are then carried freely under the carapace. This transfer of embryos to a dorsal position, using degenerating pouches as an intermediate step, may reflect how the unusual dorsal position of the brood came about during evolution. But many aspects of the origin of the unusual dorsal brooding are still uncertain, not least what kind of development was the precursor. At least two possibilities exist: (1) the dorsal brooding had its origin in another type of brooding e.g., the in peracarid-type, where embryos are brooded ventrally in a marsupium formed (probably) by modified limb epipods, or (2) the dorsal brooding had its origin in in a type of development with free larvae as that seen in euphausiids or dendrobranchiate decaopods, both of which have a relatively anamorphic development starting with free-living nauplii. Here we suggest that a transition from one specialized type of brooding (ventrally in a marsupium) to another type of brooding (in a dorsal brood pouch), is the most complicated of the two scenarios, and therefore the least likely. Both types of brooding (ventral and dorsal) are more likely to have had independent origins in a type of development with free larvae as that seen in euphasiids or dendrobranchiate decaopods. Brooding is common in Crustacea and may have many advantages. In the case of Thermosbaenacea, some of the advantages have specifically been suggested to be a consequence of a possible pelagic, bacterial feeding lifestyle, where the animals tend to be upside-down swimming [14], but there is also evidence for a more benthic lifestyle [15].

### Spine-like structures of posterior carapace margin

This study has revealed that the brooding female has rows of long, flexible spine-like structures along the posterior margin of its brood pouch (carapace) directed towards the dorsal side of the body segments (Fig 4). Their function obviously is to keep the developing embryos and later stages inside the brood pouch during development. Observations of live animals while they move their hind body alternatingly dorsally and ventrally, thereby temporarily expanding the opening of the brood pouch posteriorly (Fig 1H), have showed that the spine-like structures get bend and stretched following the movements of the body and thereby partly seals the posterior opening (Fig 1G, S4 Video). Similar spine-like structures have previously been noted for *Tethysbaena juriaani* [12]. In *Thermosbaena mirabilis* the posterior margin of the carapace is also modified for sealing the posterior opening, not as spines, but rather as a broad fold increasing the size of the carapace posteriorly [8]. Embryos brooded dorsally under a carapace are rare among crustaceans and, apart from Thermosbaenacea, found only in Ascothoracida (Thecostraca), some Ostracoda, and in Cladoceromorpha (Branchiopoda) [2]. Also in some of these taxa modifications for keeping the embryos inside the brood pouch are seen, obviously convergent from those in *Tulumella*. In *Cyclestheria hislopi* (Cladoceromorpha) the developing embryos are even attached to long, umbilical cord-like structures to the mother’s legs [26, 27] while in cladocerans such as *Daphnia* some lobate outgrowths dorsally of the abdomen serve to keep the developing embryos in place.

### Carapace, dorsal organ, eye lobes, rudimentary naupliar processes—similarities to the Cambrian Bredocaris and Rehbachiella

It is well-known that early developmental stages can be useful to consider in a phylogenetic context for several reasons. Sometimes earlier stages provide access to rudiments of structures, which were present earlier in evolution, or sometimes certain features such as external segmentation, which is of particular importance in arthropods, can be seen more clearly. The advanced developmental stages of *Tulumella unidens* examined here provide several such examples of larval or rudimentary structures, for example a dorsal organ, eye lobes, or rudimentary naupliar processes of the second antennae, and they also provide important information on early carapace development.

An enlarged carapace modified as a brood pouch in ovigerous females have been shown for a number of thermosbaenaceas such as *Thermosbaena mirabilis* and *Tethysbaena juriaani* [12], but to which extent this brood pouch is comparable to the ‘typical’ crustacean carapace (see [2]) with regard to its morphological extension and development, has been unclear. This study shows that both development and general morphology of the dorsal brood pouch in *Tulumella unidens* is virtually indistinguishable from the ‘classical’ crustacean carapace, which has been described as ‘a shield extending from the head region and enveloping a smaller or larger part of the body’ [2, 28]. In the adult ovigerous female it looks very much like the carapace of for example a leptostracan or a mysid, and its partly removal by dissection, which revealed its attachment more clearly, shows that it extends in a straightforward way directly from the rear of the cephalic portion (Fig 3B–3D), as is the case for a number of other crustaceans (see [2]). Also the development of the carapace in *Tulumella unidens*, which is as a fold growing posteriorly as well as laterally is in line with a number of other crustaceans such as in branchiopods (e.g., *Triops* and *Cyclestheria*, see [2]). A striking similarity in size and extension of the carapace is seen between the late developmental stage of *T*. *unidens* and the non-malacostracan Cambrian Orsten crustacean *Bredocaris admirabilis* [29].

The late developmental stage of *Tulumella unidens* bears a small, circular ‘dorsal organ’ at the carapace approximately in the region corresponding to where the second antennae insert ventrally (Fig 5D and 5E). It is absent in adults. A ‘dorsal organ’ has not previously been reported for the Thermosbaenacea. A number of authors have reviewed and discussed homologies of various types of ‘dorsal organs’ in both recent and fossil Crustacea and also discussed the relevance of the term ‘dorsal organ’ [18, 30–33], and this is not the place to repeat this discussion. Here we just report the presence of a ‘dorsal organ’ in the late stage of *Tulumella*. It is encircled by a cuticular border and does not bear the four sensors arranged as the corners of a square, the centre of which is occupied by a gland, which is typical for a number of dorsal organs (called ‘sensory dorsal organ’ in [31]). The smooth surface of the organ and its presence only in developmental stages seem to suggest that it is an ion transporting organ in *Tulumella* typical for embryos of many taxa [30, 31].

Adults of *Tulumella unidens* lack fully developed compound eyes but have a pair of so-called ocular scales each with a pigmented spot basally (Fig 2B). Ocular scales are well-known to be present in the thermosbaenacean genera *Halosbaena*, *Limnobaena*, *Theosbaena*, and *Tulumella*, while they are absent in *Thermosbaena* and Monodellidae [12]. Rudimentary eyes with a relatively strong innervation have been found in *Thermosbaena mirabilis*, so it is likely that they are still functional [4]. Escape response to light has been reported for *Tethysbaena juriaani*, and collecting in the field of the same species yielded distinctly more specimens from the darker part of wells compared to the parts exposed to sunlight [12]. Probably pigmented ocular scales as those reported here for *Tulumella unidens* are responsible for such response to light. The ocular scales in late stages of *Tulumella unidens* are shaped as large anterior lobes (Figs 5–7), somewhat similar to those reported for developing stages of *Thermosbaena mirabilis* [10]. The combined presence of eye lobes during development and pigmented ocular scales in adults suggest that thermosbaenaceans are less adapted for a stygobiontic lifestyle than for example Remipedia, where eyes are totally absent. The presence of eye lobes in early stages are interesting in a comparative morphological context as they resemble the eye lobes found in a number of other crustaceans, for example those seen in developing anostracans before the eyes become stalked [34], or the anterior eye lobes seen in certain late developmental stages of Cambrian crustacean fossils such as *Rehbachiella kinnekullensis* and *Bredocaris admirabilis* [18, 29].

The primordial coxae of the second antennae of the developing stages have enditic protrusions which bear incipiently developed spines/setae terminating in a tuft of sensillae (Fig 7C–7G). The position of these indicate homology to the similarly placed spine-bearing endites present in early crustacean larvae of many taxa, often termed ‘naupliar processes’, which play an active role during naupliar/larval feeding (e.g., [35–38]). The presence of such incipiently developed spines in Thermosbaenacea is remarkable as naupliar processes are absent elsewhere in Malacostraca, even in Euphausiacea and Dendrobranchiata, where free-living (but lecitotrophic) nauplii are present [37]. Contrary to other crustaceans with naupliar processes in larvae, the structures in the late stage of *Tulumella* does not serve a feeding function since mouth and other feeding structures are still not developed and the specimens are still retained in the brood pouch. They may have another function (sensory?) or they may be evolutionary rudiments. Their presence (albeit rudimentary) in Thermosbaenacea raises important evolutionary questions. First of all, these rudimentary antennal naupliar processes strongly suggest that the developmental sequence of thermosbaenaceans have been modified from an ancestor with a sequence of free-living and feeding larvae (e.g., with functional naupliar processes). But larval sequences with these characteristics are not available within Malacostraca, so the crucial question is whether the thermosbaenacean type of ‘embryonised’ developmental sequence, e.g., with rudimentary processes, (1) evolved already ancestrally in malacostracan evolution from non-malacostracan acestors, or whether it (2) evolved within Malacostraca from now non-existent taxa with nauplii of naupliar-like larvae feeding by means of an antennal naupliar process, as seen in so many non-malacostracan larvae. Considering the phylogenetic position of Thermosbaenacea as nested deeply within Malacostraca, we consider scenario (2) as the most likely.

### Club-shaped extensions of body wall in female—epipods/pleurobranchs?

Another finding for *Tulumella unidens* concerns two pairs of small club-shaped extensions/lobes dorso-laterally at the body of the female inside the brood pouch (Fig 3B–3E), which have never before been reported from any thermosbaenacean. The anterior pair—the largest—is associated with the maxilliped segment, the other pair with the second thoracic (T2) segment. Both function and morphological origin are uncertain but some possibilities will be discussed. Since the lobes are absent in non-ovigerous specimens, their function may be related to brooding. If indeed these lobes are involved in brooding they may have a simple mechanical function preventing the eggs/embryos from blocking the anterior part of the brood pouch and thereby disrupting the function of the beating respiratory epipods. Another possibility is that the club-shaped lobes have an osmoregulatory or a respiratory role. If so, it is probably unlikely that they serve in setting up a special environment for the embryos within the chamber since this is not sealed from the surroundings. Rather they may serve as gills. It is known from *Thermosbaena mirabilis* that the epipod of the maxilliped produces respiration currents which enter the carapace chamber from behind and emerges anteriorly on each side of the head, and that both the epipod itself and the inner side of the carapace has respiratory tissue [8]. The presence of a maxilliped epipod combined with observations on live animals suggests that a similar respiratory current is produced in *Tulumella unidens*. Since the two pairs of lobate extensions are placed within the female’s carapace close to where the respiratory current leaves anteriorly (Fig 3B–3E, asterisk), they would be properly positioned to serve as gills.

As mentioned, the club-shaped lobes are not directly attached to the limbs but are extensions of the lateral body wall, the ‘pleuron area’ (maxilliped lobes) or the ‘arthrodial membrane area’ (T2 lobes). Hence, if indeed these lobate stuctures are gills, then at least the anterior pair (on maxilliped segment) should be referred to as pleurobranchs and would represent the only case of gills attached to the body wall outside the Decapoda and Amphionidacea (see summary in [39]). Of much relevance in this context is a report of a first pereopod (= thoracopod 2) of an unidentified species of *Tulumella* with a simple lobate structure arising laterally from the arthrodial membrane, which was interpreted as an arthrobranch [39], which, apart from being relatively smaller, appears quite similar to the lobate structures reported here for the ovigerous specimen of *Tulumella unidens*. In the same unidentified species similar structures at thoracopods 3–5 were found [39], and single arthrobranches have also been reported for *Tulumella grandis* [40]. Due to the general similarity between these arthrobranches in other species of *Tulumella* and the more anteriorly positioned lobate structures reported here for *Tulumella unidens*, they are likely to be serial homologues, in which case those of *T*. *unidens* should also be referred to as gills.

The structural origin of the club-shaped lobes (gills?) is uncertain. They may be evolutionary new structures specific for Thermosbaenacea, or, perhaps more likely, they may have been derived from pre-existing structures, the homologues of which could be present in other crustaceans. If the latter is the case, then candidates for their derivation are epipods of which as many as two (*Tanazios*) or three (*Yicaris*) seems to be present in some recent and fossil crustaceans [41–43]. Three epipods are present in some recent anostracans [34] of which the two proximal are often referred to as pre-epipodites [39]. Three laterally placed outgrowths on the postmaxilulary limbs of the Cambrian *Yicaris dianensis* have been interpreted as epipods, as have the same number of lateral outgrowths in the Cambrian *Paulinecaris siveterae* [41–43]. Such derivation of gills from epipodites within Malacostraca (especially Decapoda) was suggested long ago by (e.g., [44–46]). The general understanding is that the more distal gills (e.g., podobranchs) would have been derived from the distal epipodite, while the more proximal gills (e.g., arthrobranchs and pleurobranchs) would have been derived from more proximal epipods (‘pre-epipodites’). If indeed the proximal epipods seen in the mentioned Cambrian crustaceans and anostracans are the homologues to the more dorsally placed gills in malacostracans, such as the putative pleurobranchs/arthrobranchs reported here for *Tulumella unidens*, it would require a migration (or another process with similar result) of such structures from a position laterally at the limbs as that seen in anostracans and the mentioned Cambrian Crustaceans to a position laterally at the body wall as seen in the mentioned malacostracans (including *Tulumella*).

The situation concerning epipods in malacostracans is quite complex. There is much variation serially between appendages in the epipodal armature, and much variation between taxa, and, complicating the situation even further, there has been much debate on the eventual epipodal nature of structures such as östegites (in peracarids), coxal plates (in amphipods), and gills (in decapods and others) (see review in [39]). Within Malacostraca only Anaspidacea has pereopods with two lateral structures attached, which, because of their position laterally on the limbs and their rounded shape, uncontroversially have been termed epipods. In the Silurian *Cinerocaris magnifica*, which has a strong affinity to the Leptostraca [47], two epipods may be present (in contrast to one in recent Leptostraca), which may be indicative of the ancestral malacostracan condition. For *Tulumella unidens* the discovery of putative epipods associated with thorax segments 1 and 2 (Fig 3B–3E) provides additional information to the ongoing discussion of the number of epipods and the nature of these in the Malacostraca. If indeed these structures are epipods, for which we see most evidence, then *Tulumella unidens* has not only one but two epipods associated to the maxilliped (the lobate structure reported here and the ‘normal’ epipod).

### Homologies of cephalic appendages in Thermosbaenacea to those of other Crustacea (Figs 8 and 9)

**Fig 8:**
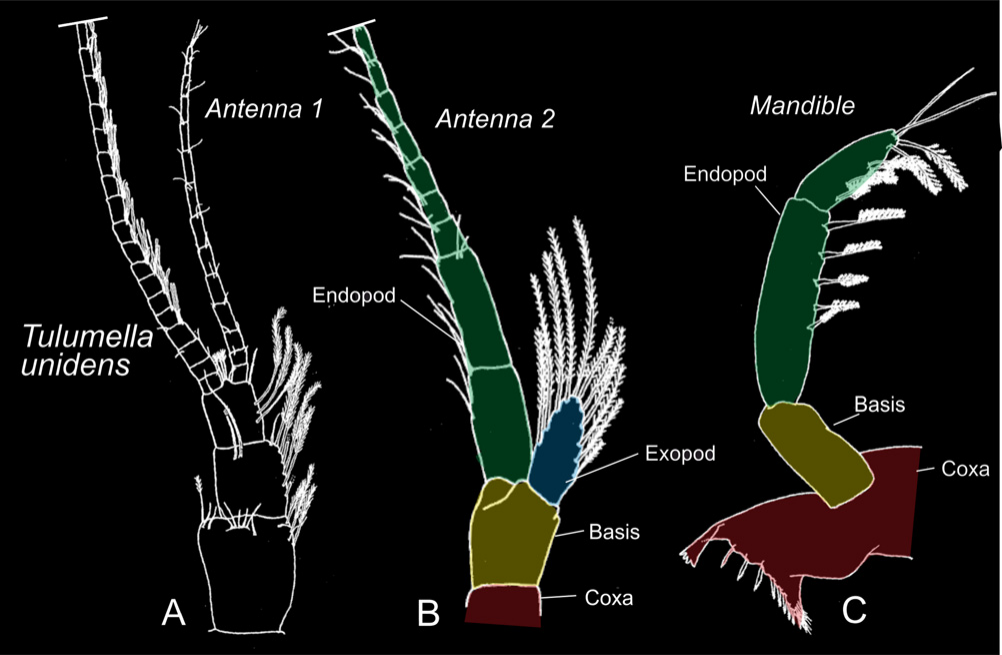
Antenna 1, 2, and mandible of *Tulumella unidens* (Thermosbaenacea), with presumed homologous structures indicated by same colour code: Red-coxa, yellow-basis, green-endopod, blue-exopod, purple-epipod. Compiled and modified from Wagner (1994).

**Fig 9:**
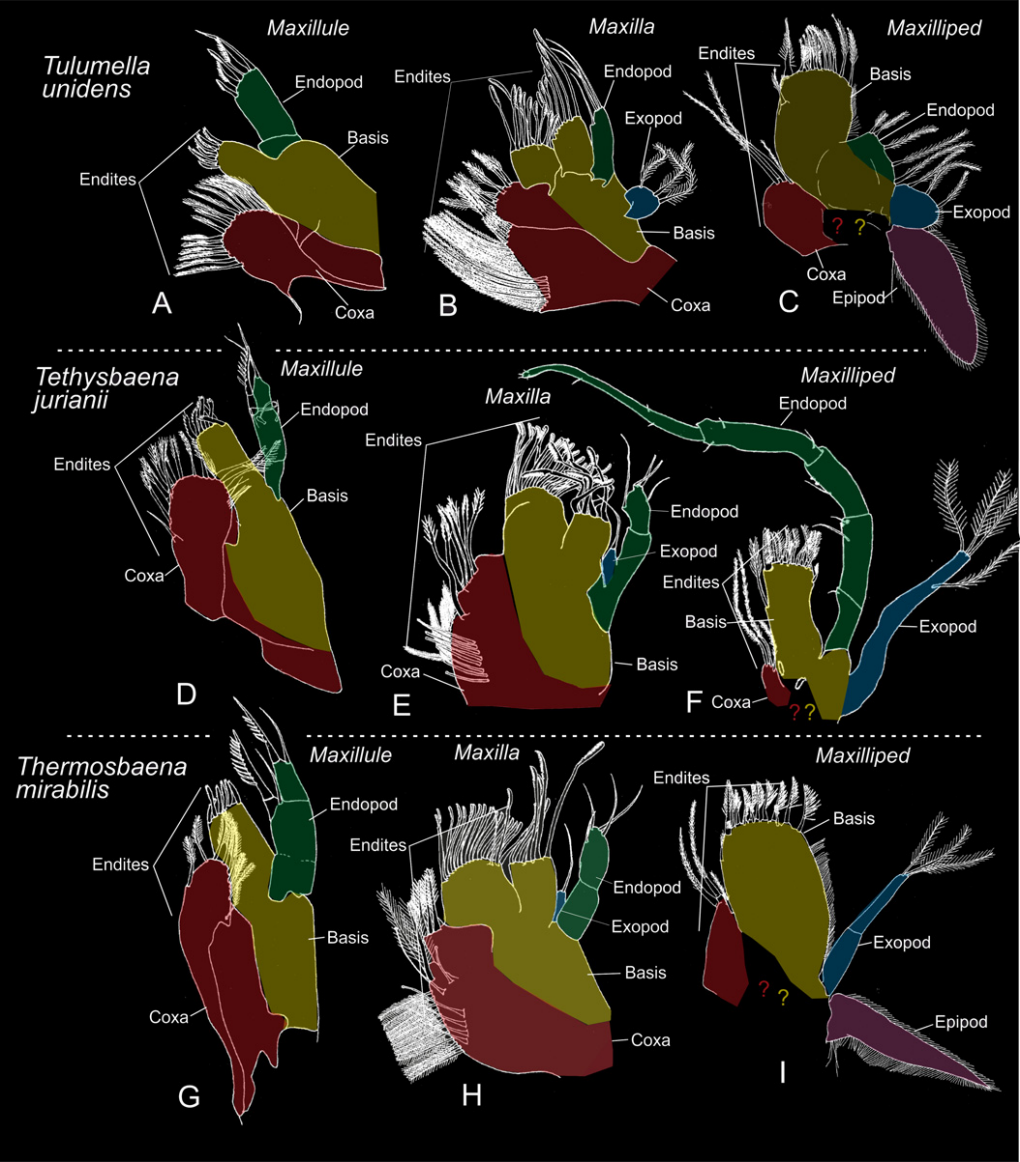
Certain mouth parts (maxilla 1, 2, and maxilliped) of three species of the Thermosbaenacea covering most variation, with presumed homologous structures indicated by same colour code: Red-coxa, yellow-basis, green-endopod, blue-exopod, purple-epipod. Compiled and modified from Wagner (1994).

Some uncertainty exist concerning the precise homologies of thermosbaenacean mouth-parts to those of other crustaceans [12, 15], which is probably due to a quite modified feeding mode involving scraping and brushing by these limbs (see [15]). Building on the assumption that earlier developmental stages (embryos, larvae) sometimes provide a view into an earlier evolutionary period of a taxon or just shows e.g., segmentation patterns more clearly, we attempt to apply conventional crustacean limb terminology such as ‘coxa’, basis’, etc. to cephalic limbs of *Tulumella unidens* (see Figs 8 and 9, colour code corresponds to previous figures), and also analyse to which extent this species can be seen as a typical representative of the Thermosbaenacea. This may prove important for further clarification of the phylogenetic affinity of Thermosbaenacea on a morphological basis.

In the first antennae of the advanced developmental stages of *Tulumella unidens* basically the same components as in the adult can be recognised (Fig 8A) [12, 24], which is a 3-segmented peduncle continuing distally into an inner (shorter) and an outer (longer) branch (Figs 5, 6A, 6C and 7A). The terms ‘endopod’ and ‘exopod’ has previously been used for these branches [12], but is now well-recognised to be inappropriate since such branches in the Malacostraca are independent from—and therefore non-homologous to—the biramous (endopod/exopod) morphology of post-antennulary limbs in Crustacea in general (e.g., [16, 18]).

The morphology of the second antennae in *Tulumella unidens* has not been fully understood previously, at least not in the context of general crustacean morphology. In the adult it has been briefly described previously and the presence of a characteristic ‘scale’ laterally was noted [24], which is diagnostic for the genus *Tulumella*, lacking in all other thermosbaenaceans (Fig 8B). The second antennae of the advanced developmental stages of *T*. *unidens* consist of a coxa, basis, and two rami (endopod, exopod) (Figs 5A, 5C, 5E, 6A, 6C, 7A, 7E and 7F), which are essentially the same components as seen in a number of larval or non-malacostracan crustaceans. The proximal portion of the limb is easily identified as the coxa by the presence of a primordial ‘naupliar process’ (coxal endite) medially (see above) (Fig 7D and 7E), which is always coxal when this is present in free-living crustacean larvae (e.g., [37]). Distal to the coxa is the basis, which is separated from the coxa, at least laterally, by a weak suture. The basipodal nature of this limb portion is recognised by the presence of an ‘antennal’ scale at its disto-lateral corner, which is certainly the unsegmented, scale-like exopod (Fig 6C). The basis continues into a many-segmented endopod (Fig 6C). In the light of this developmental information, the morphology of the adult second antennae in Thermosbaenacea can be understood better. In the adult the second antenna is consistently referred to as composed of a ‘5-segmented peduncle’ with an ‘antennal scale’ attached to the ‘third peduncular segment’ [12], but since the available drawings in literature only shows two segments proximal to the antennal scale (Fig 8B) [12, 24], and since we have found only two segments in the advanced developmental stages (coxa and basis), we assume that two is the true number, which yield a peduncle consisting of four segments, the two proximal of which are the coxa and the basis (Fig 8B). Hence, in this case the coxa and the basis are not equivalent to the penduncle, which includes more limb portions. In other thermosbaenaceans the second antennae are uniramous and bear no exopodal scale and the segments decrease regularly in size from proximal [12] so a clear distinction between a ‘penduncle’ and ‘flagellum’ is not possible, but, assuming homology to *Tulumella unidens*, the two proximal segments in antenna 2 of these other taxa most likely are the coxa and the basis.

The mandible in the adult of all thermosbaenaceans is often described as consisting of a basal, swollen part carrying a molar, an incisor, often a ‘lacinia mobilis’, and distally a three-segmented palp (Fig 8C) [12]. This type of mandible morphology is widespread within malacostracan crustaceans, and the uniramous morphology often considered a malacostracan synapomorphy. These parts can be easily identified in the advanced developmental stages of *Tulumella unidens* examined here, but in a primordial developmental stage (Figs 6C and 7). Based on a comparison with antenna 2, where the coxa, basis, endopod, and exopod could be identified with high degree of confidence, and assuming some degree of serial homology with the mandible, general crustacean limb terminology can be applied to the mandible of *Tulumella unidens*, and, by extension, to other thermosbaenaceans. The proximal part, which carries the gnathal edge in the adults [25], and which is already rather extended medially into and endite-like process in the late stage (Fig 7E and 7F) is the coxa; the other possibility would be that this part of the limb should be a fusion product between the coxa and the basis, but we have found no ontogenetic evidence for that. The mandibular ‘palp’ in the late developmental stage of *T*. *unidens* consists of three portions (3 segments in adults) and is quite similar in general morphology (but shorter) to the basis and endopod of antenna 2 (Fig 7G), and, based on this similarity, which is an argument for serial homology, we assume that the palp consist of a basis (proximal palp segment) and two endopodal segments. This general morphology of the mandible is the same in all thermosbaenaceans [12], and also in many other malacostracans, which suggest that the same terminology can be applied (coxa, basis, and two endopod segments).

The first maxilla in all species of Thermosbaenacea consists basically of the same components: two endites and a 2-3-segmented palp (using terminology from [12], see also Fig 9A, 9D and 9G). The present study of the first maxilla of a late stage of *Tulumella unidens*, where the same components as in the adult can be found, reveals that the proximal of the two endites, together with the corresponding proximal part of the limb bud, is separated from the more distal part of the limb bud by a relatively distinct suture circumscribing the limb (arrows on Fig 7F and 7G). The presence of this suture suggests that the proximal part of the limb is a coxa followed by a basis, which medially extends into an endite. If serial homology is assumed, the presence of a coxa and basis is supported by comparison to the more anterior limbs (antenna 2 and mandible), where the same limbs portions are found (compare a2, md, mx1 in Fig 7F and 7G). The distal ‘palp’, which is only weakly divided into two portions in the examined late stage, is considered to be the endopod due to a position on the limb similar to that seen for what is certainly the endopod in the second maxilla (see below). A general pattern of the first maxilla as the one seen in *Tulumella unidens* and in other thermosbaenaceans is common in Malacostraca with some variation (not to be treated here) found in for example leptostracans, decapods, and many peracarids such as cumaceans, tanaidaceans, many amphipods (e.g., [48–50]) and some anaspidaceans [51]. Basically the same pattern is present in euphausiids, but here with an exopod attached to the more distal of the endite-bearing limb portions (e.g., [52]), indicating that this is the basis, as assumed here for *Tulumella unidens* and other Thermosbaenacea. A first maxilla consisting of endite-bearing coxa and basis with lateral, flaplike exopod and a (segmented?) endopod is likely to be plesiomorphic for Malacostraca. In many peracarids such as isopods, mysids, stygiomysids, bathynellaceans, ‘mictaceans’, and spelaeogriphaceans, the first maxilla only consists of the coxa and basis, which is probably the derived condition, but the phylogenetic significance needs clarification. In relation to the phylogenetic position of the Thermosbaenacea, it is of interest to note that the gross morphology of the first maxilla in itself does not support a position close to spelaeogriphaceans or ‘mictaceans’.

The second maxilla within Thermosbaenacea has a rather varied morphology (see examples in Fig 9B, 9E, 9H and [12]) and certain parts are not straightforward to homologise to other malacostracans. The second maxilla in *Tulumella* is the simplest one to understand in a wider crustacean context. In the adult it is a broad, flattened structure with a number of marginal lobes (Fig 9B), the identity as either endites, endopod, or exopod can be identified relatively easily based on evidence from a late ontogenetic stage and on comparison with other malacostracans. The lateral lobe on maxilla 2 is the exopod indicated by its lateral position, unsegmented appearance, and marginal setation, similar to what is seen in a number of other malacostracans (e.g., mysids). In contrast, it is not straightforward to identify the exopod of the second maxilla of other genera of the Thermosbaenacea. Probably it is represented by a small lobe in the vicinity or even attached to the endopod in *Thermosbaena* and species of *Tethysbaena* (see Fig 9E, 9H and [12]). Being located most often on the ‘wrong’ side (= not lateral) of the endopod, some evolutionary modification will then have to be assumed. The type of exopod seen in *Tulumella* probably is closest to the plesimorphic condition in Thermosbaenacea (outgroup comparison). The endopod in *Tulumella unidens* is represented by the slender, unsegmented lobe at the latero-distal corner of the limb (Fig 9B). Its identity as an endopod is evidenced by its serial similarity in shape and position in the limb bud of an advanced developmental stage to what is certainly the endopod of the first maxilla in the same stage (Figs 6C and 7F). Species of *Tulumella* are the only thermosbaenaceans to have an unsegmented second maxilla endopod; all other species have a two-segmented endopod [12], which is probably plesiomorphic since this is seen in for example mysids, lophogastrids, and stygiomysids [52–54]. Along the distal and inner margin of the second maxilla in *Tulumella unidens* is a total number of four lobes normally categorised as endites, the two distal of which are directed distally and carrying rows of distinct scraper setae (Fig 9B). This enditic arrangement is basically the same in all thermosbaenaceans. Among workers providing a statement on the status of these endites regarding homology to other malacostracans, two possibilities exist. Some refer to only the basal-most endite as being coxal, and the three more distal endites as being basipodal [12] but another possibility is that the *two* basal-most endites are coxal and the two following endites basipodal. Regarding this question, we note that in late developmental stages of *T*. *unidens* the two basal-most ones are weakest separated from each other, which may indicate that they belong to the same limb portion, which would be the coxa. Located between these putative coxal endites and the endopod are two endites, which are more distinct, which would then be basipodal endites. In all other thermosbaenaceans the arrangement of second maxilla endites are basically the same (Fig 9E and 9H and Wagner 1994). Regarding other malacostracans, second maxillae with four median endites are seen in taxa such as leptostracans and euphausiaceans (e.g., [52, 55]). In the case of euphausiaceans, the two proximal endites belong to a distinct coxal portion, the two distal to a distinct basipodal portion, from which the condition in thermosbaenaceans would be easily derivable.

The maxilliped of *Tulumella unidens* is unusual both in a thermosbaenacean and peracarid context since it lacks distinct and segmented endopod and exopod. In this respect *T*. *unidens* seems to be among the most derived thermosbaenaceans. Indeed, none of these structures are straight-forward to locate in the adult of *T*. *unidens* (see Fig 9C), but their identity are rather easily revealed in an advanced developmental stage based on positional serial similarity to the early lobes of what certainly will become the endopod and exopod of the second maxilla (compare these limbs in Fig 6D). In males of *Tethysbaena* the endopod is long and composed of five segments (e.g., Fig 9F and [12]), and, as this is a very widespread type of morphology within peracarids, it is most likely the plesiomorphic condition within Thermosbaenacea. Also the exopod is rather long in a number of other thermosbaenaceans, which is in contrast to the situation in *Tulumella* where it is a small, rounded structure (Fig 9C). In other peracarids the structure of the maxillipedal exopod—when this limb part is present—is rather varied. Among three potential candidates to archaic looking peracarids, three different types of maxillipedal exopods are seen: (1) a long, flagellate, many segmented structure is seen in mysids (e.g., [54]), bearing some resemblance to certain thermosbaenaceans (e.g., Fig 9F and 9I), (2) a small, lobate structure in stygiomysids [53] quite similar to that seen in *Tulumella unidens* (Fig 9C), (3) or something in between these two types as seen in lophogastrids [52]. This variation in morphology outside the Thermosbaenacea makes it difficult to identify the ancestral state within the Thermosbaenacea, and further analysis is beyond the scope of this work. The maxilliped of *Tulumella unidens* bears a large, dorsally directed lobate epipod as has been shown for a number of other thermosbaenaceans (e.g., *Thermosbaena mirabilis*). However, for many species an epipod is not illustrated nor mentioned [12], but it is uncertain to which extent this just reflects lack of information. The next part of the maxilliped to be discussed is the median side. In all thermosbaenaceans this side of the limb is composed of two endites, a large distal one and a smaller proximal one, both of which can be easily identified in the advanced developmental stages of *Tulumella unidens* (Fig 7G). The distal of these is certainly basipodal since the endopod is attached right next to it; the more proximal and smaller endite therefore is most likely coxal. The large basipodal endite as seen in thermosbaenaceans is a very characteristic structure and is a ‘typical’ peracarid feature found in a larger or smaller version in most peracarids.

### Conclusions with comments on phylogenetic position of Thermosbaenacea

The aim of this work was to study brooding and developmental aspects of a species of the Thermosbaenacea in a phylogenetic context. A number of new morphological structures have been reported and some already known aspects re-addressed by new methods, some of which may have phylogenetic relevance for placing the Thermosbaenacea within the Malacostraca. However, it has been beyond the scope of this work to provide a complete analysis of a more complete set of characters relevant for placing the Thermosbaenacea phylogenetically.

Some main results:

- Dorsal brooding is confirmed for yet another species of the Thermosbaenacea, the first time for the genus *Tulumella*. This for malacostracan crustaceans unusual brooding habit is considered incompatible with an ingroup position within the Peracarida, since peracarids exhibits another type of highly modified brooding in a ventral marsupium. This is in line with previous suggestions [4, 20].
- A ventrally flexed hind body of advanced developmental stages is seen in *Tulumella unidens* and in embryos of other thermosbaenaceans and is probably plesiomorphic [20]. A dorsally flexed hind body, which is most likely apomorphic, is found in Cumacea, Isopoda, Mictacea, Tanaidacea, Speleaogriphacea, so, based on this character alone, Thermosbaenacea would be placed outside this assemblage.
- Rudimentary antennal naupliar processes in a late developmental stage suggest that the development sequence of Thermosbaenacea have evolved from a larval sequence with free nauplii or nauplius-like larvae with active feeding appendages (incl. naupliar processes).
- The morphology of the carapace (brood pouch) in an ovigerous female and its development in a late stage is in accordance with the ‘classical’ crustacean carapace.
- The late stage exhibits a larval dorsal organ similar to that found in many other recent and fossil crustaceans, both larvae and adults.
- The combined presence of eye lobes during development of *Tulumella unidens* and pigmented ocular scales in adults suggest that thermosbaenaceans are less adapted for a stygobiontic lifestyle than for example Remipedia, where eyes are totally absent.
- The ovigerous female bears two pairs of small club-shaped extensions/lobes dorso-laterally at the body inside the brood pouch. It is suggested that these are modified epipods possibly serving as gills or preventing the eggs/embryos from blocking the anterior part of the brood pouch and thereby disrupting the function of the beating respiratory epipods.
- An analysis of limb segmentation of late developmental stages of *Tulumella unidens* resulted in a complete hypothesis for thermosbaenacean limb homologies to other Crustacea (see details above).

## Supporting Information

S1 Video Ovigerous female of *Tulumella unidens* (Thermosbaenacea) filmed in petri dish in lab. (AVI)

S2 Video Advanced developmental stages of *Tulumella unidens* (Thermosbaenacea) moving within the female’s brood pouch. (AVI)

S3 Video Ovigerous female of *Tulumella unidens* (Thermosbaenacea) with advanced developmental stage in brood pouch in the process of becoming released. (AVI)

S4 Video Ovigerous female of *Tulumella unidens* (Thermosbaenacea) showing brood pouch with posterior sealing mechanism (long marginal spines). (AVI)

S5 Video Newly released advanced developmental stage of *Tulumella unidens* (Thermosbaenacea). (AVI)
