# Correction: The Unique Dorsal Brood Pouch of Thermosbaenacea (Crustacea, Malacostraca) and Description of an Advanced Developmental Stage of *Tulumella unidens* from the Yucatan Peninsula (Mexico), with a Discussion of Mouth Part Homologies to Other Malacostraca

**DOI:** 10.1371/journal.pone.0133109

**Published:** 2015-07-15

**Authors:** The PLOS ONE Staff

## Notice of Republication

This article was republished on June 23, 2015, to correct Figs 3 and 4 in the online version, which were darker than intended. The publisher apologizes for the error. Please view this article again to see the corrected figures.
